# Structural and Functional Motifs in Influenza Virus RNAs

**DOI:** 10.3389/fmicb.2018.00559

**Published:** 2018-03-29

**Authors:** Damien Ferhadian, Maud Contrant, Anne Printz-Schweigert, Redmond P. Smyth, Jean-Christophe Paillart, Roland Marquet

**Affiliations:** CNRS – UPR 9002, Architecture et Réactivité de l’ARN, IBMC, Université de Strasbourg, Strasbourg, France

**Keywords:** influenza, influenza A virus, RNA, RNA structure, promoter, vRNA, CRNA

## Abstract

Influenza A viruses (IAV) are responsible for recurrent influenza epidemics and occasional devastating pandemics in humans and animals. They belong to the *Orthomyxoviridae* family and their genome consists of eight (-) sense viral RNA (vRNA) segments of different lengths coding for at least 11 viral proteins. A heterotrimeric polymerase complex is bound to the promoter consisting of the 13 5′-terminal and 12 3′-terminal nucleotides of each vRNA, while internal parts of the vRNAs are associated with multiple copies of the viral nucleoprotein (NP), thus forming ribonucleoproteins (vRNP). Transcription and replication of vRNAs result in viral mRNAs (vmRNAs) and complementary RNAs (cRNAs), respectively. Complementary RNAs are the exact positive copies of vRNAs; they also form ribonucleoproteins (cRNPs) and are intermediate templates in the vRNA amplification process. On the contrary, vmRNAs have a 5′ cap snatched from cellular mRNAs and a 3′ polyA tail, both gained by the viral polymerase complex. Hence, unlike vRNAs and cRNAs, vmRNAs do not have a terminal promoter able to recruit the viral polymerase. Furthermore, synthesis of at least two viral proteins requires vmRNA splicing. Except for extensive analysis of the viral promoter structure and function and a few, mostly bioinformatics, studies addressing the vRNA and vmRNA structure, structural studies of the influenza A vRNAs, cRNAs, and vmRNAs are still in their infancy. The recent crystal structures of the influenza polymerase heterotrimeric complex drastically improved our understanding of the replication and transcription processes. The vRNA structure has been mainly studied *in vitro* using RNA probing, but its structure has been very recently studied within native vRNPs using crosslinking and RNA probing coupled to next generation RNA sequencing. Concerning vmRNAs, most studies focused on the segment M and NS splice sites and several structures initially predicted by bioinformatics analysis have now been validated experimentally and their role in the viral life cycle demonstrated. This review aims to compile the structural motifs found in the different RNA classes (vRNA, cRNA, and vmRNA) of influenza viruses and their function in the viral replication cycle.

## Introduction

**FIGURE 1:**
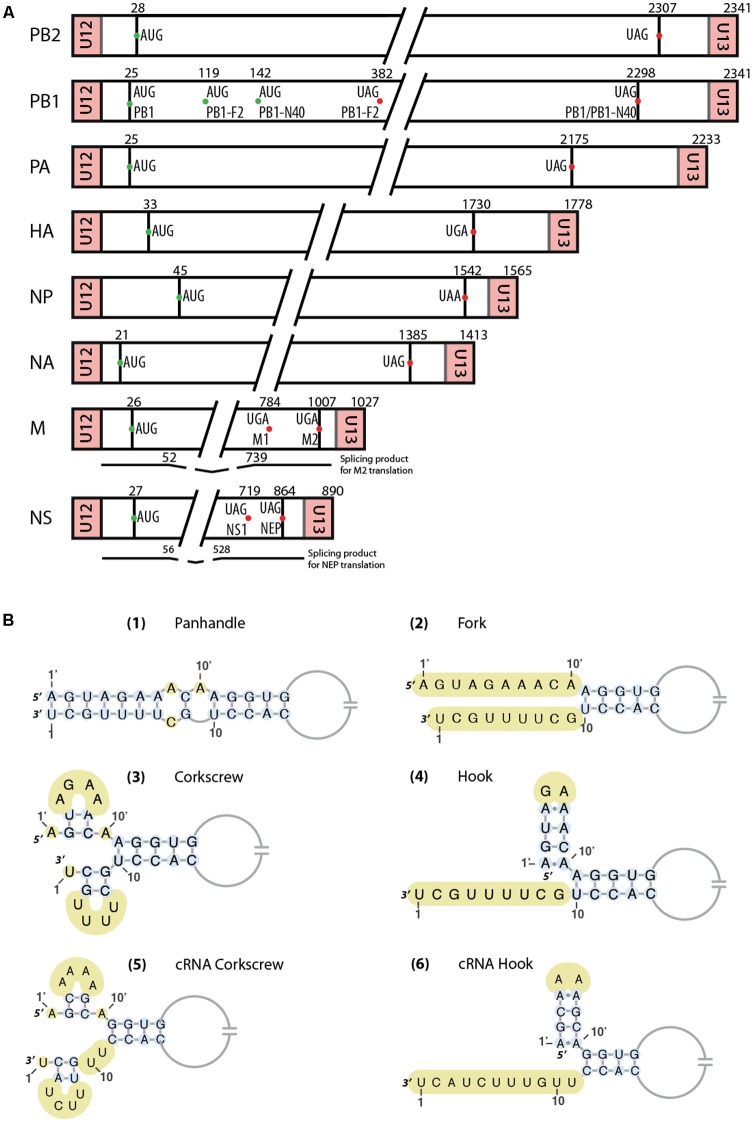
**(A)** Schematic representation of the eight RNA segments of Influenza A viruses. AUG start codons of the different proteins are represented as green dots, while stop codons are indicated by red dots. Regions between U12/U13 and start codons correspond to the untranslated regions (UTRs). Below the M and NS segments are represented the spliced mRNAs. Important nucleotides positions are indicated. **(B)** Different vRNA promoter structure models proposed over time. Unpaired nucleotides are highlighted in yellow. In the ‘hook structure’ (**4,6**), two canonical base-pairs are flanked by two non-canonical A-A base-pairs.

Influenza viruses belong to the *Orthomyxoviridae* family that comprises seven genera: *Alphainfluenzavirus*, *Betainfluenzavirus*, *Gammainfluenzavirus*, and *Deltainfluenzavirus*, as well as *Isavirus* (which infects salmon), *Thogotovirus* (transmitted by arthropods), and *Quaranjavirus* (which predominantly infects insects and birds). Among this family, the major threat for public health is the *Alphainfluenzavirus* genera. Influenza B and C share common ancestry with *Influenza A virus* (IAV). However, *Influenza B* (IBV) and *C* (ICV) *viruses* mainly infect human and are genetically less diverse. IBV, like IAV, has 8 genome segments coding for at least 11 proteins (**Figure 1A**), whereas Influenza C has only 7 genome segments coding for at least 9 proteins. The segments are either numbered from the longest to the shortest or named after the main protein they code for, e.g., in influenza A viruses, segments 1–8 are also named PB2 (Polymerase Basic 2), PB1 (Polymerase Basic 1), PA (Polymerase Acid), HA (HemAgglutinin), NP (nucleoprotein), NA (NeurAminidase), M (Matrix), and NS (Non-Structural), respectively.

Each segment constitutes an individual replication unit coding for at least one essential protein and form viral ribonucleoproteins (vRNPs). They are composed of a viral RNA (vRNA) of negative polarity, a heterotrimeric polymerase complex bound at the paired 5′ and 3′ termini of the vRNA and multiple copies of NP (Eisfeld et al., 2015).

After viral entry in the host cell, vRNPs are imported in the nucleus, where transcription and replication take place. During these steps, two different classes of RNA of positive polarity are synthetized: complementary RNA (cRNA), which is the exact copy of vRNA, and viral messenger RNA (vmRNA) (Eisfeld et al., 2015). The latter one is synthetized using a capped primer obtained by cap-snatching, a mechanism during which capped cellular mRNAs are recognized and cleaved by the polymerase complex (Eisfeld et al., 2015). On the contrary, cRNA are synthetized *de novo* and are used as template for producing new vRNA molecules that will be packaged.

The RNA secondary structure is biologically important for many RNA viruses, governing key processes, including viral replication, RNA packaging, RNA editing, and mRNA splicing regulation. Influenza viruses are no exception and several mechanisms during their life cycle are controlled by RNA structure. Steps such as splicing, the switch between transcription and replication, vRNA packaging and recognition by the host immune system have been proposed to be under the control of RNA structure. Only few things are known about the structure of the vRNAs and even less about the structure–function relationships, but things are starting to change dramatically thanks in part to new methodologies exploiting the potential of next generation RNA sequencing.

This review aimed to compile the structural motifs found in the different RNA classes of influenza viruses and their function in the viral replication cycle. A previous review was published many years ago and focused on the differences and similarities between the RNA structure in IAV and the genome structure of other RNA viruses (Gultyaev et al., 2010).

## Influenza a Virus

### Negative Polarity RNA (vRNA)

#### The Terminal Promoter

For convenience, in this review, the numeration of nucleotides forming the promoter will be as follows: from the 5′ terminus, nucleotides will be numbered as X′, while from the 3′ terminus, they will be numbered as X.

##### Historical background

**FIGURE 2:**
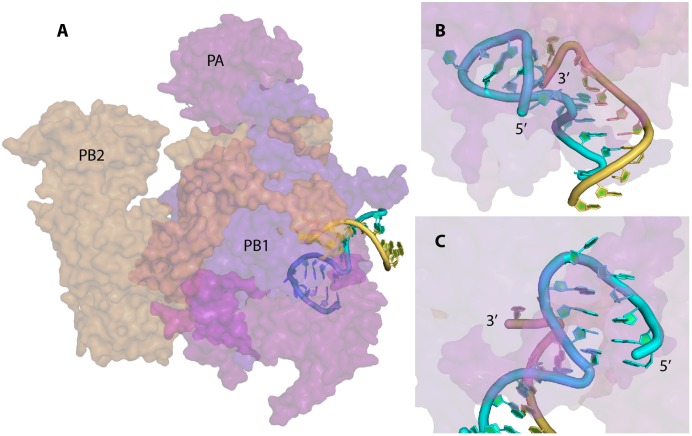
X-ray structure of the vRNA promoter bound to the polymerase complex of bat IAV. The surface of the PA, PB1, and PB2 subunits are represented in magenta, violet-blue, and orange, respectively; the 5′ and 3′ strand of the vRNA promoter are drawn in cyan and yellow, respectively. This figure was drawn with PyMOL (The PyMOL Molecular Graphics System, Version 2.0 Schrödinger, LLC.) using the coordinates obtained by Pflug et al. (2014) (Protein Data Bank accession number 4WSB). **(A)** General view of the complex. **(B)** and **(C)** Close up views of the promoter and the stem loop formed at the 5′ end of the 5′ strand. The 5′ end of the 5′ strand and the 3′ end of the 3′ strand of the promoter are labeled.

**FIGURE 3:**
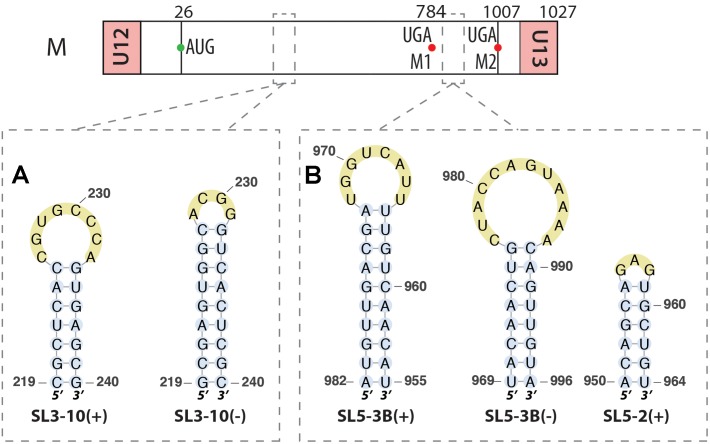
RNA structures identified along the segment M in both (–) and (+) strands. Their locations are indicated by dashed squares on the segment scheme at the top of the figure. **(A)** Similar stem-loop SL3 found in both strands at the exact same positions. **(B)** Other stem-loops found in this segment by bioinformatics analysis.

The structure of the vRNA promoter has been a controversial topic in the literature, and as many as four different models have been proposed (**Figure 1B**). They all share some features such as base pairing between certain parts of the 3′ and 5′ ends but are quite different when it comes to the structure of the first nucleotides at each end. Hsu et al. (1987) observed that the vRNA formed circular structures after light induced-RNA crosslinking with psoralen and proposed that these structures are the result of a panhandle structure (**Figure 1B-1**) arising from sequence complementarity between the 3′ and 5′ vRNA ends. They proposed that this panhandle structure would be found only within vRNPs, and that no interaction between the two ends would take place in ‘naked’ vRNAs. The same year, Honda et al. (1987) also proposed base pairing between the 3′ and 5′ ends of the vRNA in their structural model of the RNP core. In contrast, based on RNA probing experiments, Baudin et al. (1994) proposed that within the panhandle, the 3′ and 5′ ends are not paired (**Figure 1B-2**). Such a pairing would occur in naked vRNAs but the addition of NP would melt the structure. The same year, Fodor et al. (1994) kept the idea of a pairing between the two ends forming the promoter but proposed the ‘fork model’ for the transcription initiation. In this model, base pairing takes place from nucleotide 13 at the 3′ end and 11′ at the 5′ end, while the vRNA ends are single stranded (Fodor et al., 1994). Flick et al. (1996) proposed a third model called ‘corkscrew model’ (**Figure 1B-3**). In this model, two short stem-loops are formed within the 3′ (nucleotides 1–9) and 5′ ends (nucleotides 1′ to 9′) and a five base-pair ‘duplex region’ is formed between both ends from nucleotides 11′ and 10 to nucleotides 13′ and 12 (Flick et al., 1996). Moreover, it was proposed that conservation of nucleotides at the 3′ and 5′ ends could be extended by up to 4 nucleotides in a segment-specific way (Desselberger et al., 1980), thus allowing an extension of the duplex region of the promoter. An important structural feature observed by Flick et al. (1996) in this promoter is that nucleotide A10′ needs to be unpaired and forms a flexible junction. When a U residue was inserted in the 3′ end to form a base pair with A10′, transcription was impaired (Flick et al., 1996). The unpaired nucleotide A10′ is found in all the proposed structures, except for the one from Hsu et al. (1987) where it is nucleotide A11′ that is unpaired. However, using a reporter assay, Flick et al. (1996) showed that A11′ is paired with U10 and A10′ is unpaired. The functions of this specific structural feature will be discussed below.

##### Crystallographic structure of the polymerase complex and functional implications

**FIGURE 4:**
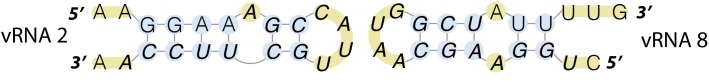
Kissing-loop interaction from between segments PB1 and NS of an avian H5N2 virus. These regions were identified to interact with each other by electrophoretic mobility shift assay and the folding of these structures suggests an interaction via a kissing-loop mechanism.

The vRNA promoter of IAV is used for two main mechanisms, transcription (which leads to the synthesis of capped and polyadenylated vmRNA) and replication (synthesis of cRNA which will be used as template for vRNA synthesis). The fork model described earlier was proposed in order to explain the initiation of the transcription, where the polymerase complex first binds the single-stranded 5′ end of the vRNA and then the single-stranded 3′ end (Fodor et al., 1995). However, a recent crystal structure of the vRNA promoter bound to the polymerase complex of bat IAV showed that this is not the conformation adopted by the promoter (Pflug et al., 2014). In this complex, the promoter adopts a structure similar to the ‘corkscrew’ model, except that only the 5′ strand forms a stem-loop, while the 3′ strand is single stranded and enters into the polymerase active site (**Figures 1B-4**, **2**). The 5′ stem-loop (or ‘hook’) is formed by two canonical base-pairs flanked at both sides by non-canonical A-A base pairs, and this compact structure binds in a pocket at the interface of the PA and PB1 polymerase subunits (Pflug et al., 2014). These results are in agreement with the literature (Hsu et al., 1987; Baudin et al., 1994; Fodor et al., 1994), as it seems that the promoter folds as a panhandle (**Figure 1B-1**) only in the absence of the polymerase. Accordingly, Noble et al. (2011) showed using biophysical analysis that in solution and in the absence of polymerase complex the 5′ and 3′ vRNA ends adopt a panhandle structure. Kierzek’s team reached the same conclusion by performing RNA chemical probing on ‘naked’ full-length NS and M vRNAs (Lenartowicz et al., 2016; Ruszkowska et al., 2016). However, the general consensus is that the IAV promoter is never free during the viral life cycle, and thus the biological function of this extended panhandle structure (**Figure 1B-1**) is unclear. When the polymerase complex is bound to the promoter, a structural rearrangement occurs with the formation of a hook in the 5′ end proximal region, leaving the 3′ end single stranded (Pflug et al., 2014). The distal region of both strands remains unchanged and forms a RNA duplex between both strands (**Figure 2**).

As mentioned earlier, the duplex region of the promoter can be extended in a segment-specific way. In the case of the HA segment, this duplex can be extended by forming a base pair between nucleotides 13 and 14′. Wang et al. (2016) analyzed the functional importance of nucleotides 13–15 and 14′–16′ (where only nucleotides 13–14′ form a base-pair). They found that the 13–14′ base pair of the duplex is important for the promoter activity, that base-pairing between nucleotides 14 and 15′ enhances this activity, and that nucleotides 15 and 16′ do not play any role in promoter activity. Importantly, mutations of the 13–14′ base pair and of nucleotide 14 showed a reduction of packaging efficiency not only of the HA segment but also of the PB1, PB2, and PA segments (Wang et al., 2016). This strongly suggests that the segment-specific duplex extension is involved in transcription/replication and, at least in one case, in vRNA packaging.

Another step of the transcription mechanism that implicates the promoter structure is the endonuclease activity of the polymerase complex. This activity is involved in the cap-snatching mechanism, where the polymerase complex ‘steals’ the 5′ capped end of cellular mRNAs that will be used as primer for the viral mRNA synthesis. Leahy et al. (2001a) determined the importance of the structure of the 5′ and 3′ ends for the endonuclease activity. They showed, using mutagenic analysis, that the 5′ stem of the corkscrew model is essential for this activity as well as the RNA duplex formed by the association of the 3′ and 5′ strands (**Figure 1B-3**) (Leahy et al., 2001a). Of note, these structural elements also exist in the hook structure observed in the crystal structure of the promoter bound to the polymerase (**Figure 1B-4**) (Pflug et al., 2014). Using the same approach, Leahy et al. (2001b) showed that some mutations in the 3′ end disrupted the endonuclease activity, suggesting the presence of a 3′ hairpin loop. They proposed a model in which the polymerase complex binds the 5′ hairpin loop and induces a conformational change to form the 3′ hairpin loop that will be needed for the cap-snatching mechanism. Importantly, comparison of the X-ray structure of the polymerase complexes of IAV and IBV suggests that capped snatching requires rotation of the PB2 cap-binding domain to direct the capped primer first towards the endonuclease domain in the PA subunit and then into the polymerase active site located in the PB1 subunit (Reich et al., 2014).

During viral transcription, polyadenylation of vmRNAs is also regulated by the vRNA promoter structure. Indeed, Luo et al. (1991) showed that the promoter needs to be structured (3′ and 5′ end paired) to allow the stuttering of the polymerase on the uridine stretch that is found 15–17 nucleotides from the 3′ end, and hence addition of the polyA tail to vmRNA by the transcribing viral polymerase. Indeed, template translocation is likely prevented by tight binding of the 5′ hook to the polymerase (Pflug et al., 2017).

The results discussed above show that the vRNA promoter structure is essential for the whole transcription process, from the beginning (cap-snatching) to the end (polyA tailing). In addition, the switch between transcription and replication of the vRNA could also be due, at least partly, to the promoter structure. Indeed, even though the IAV promoter is highly conserved across viral strains and genomic segments, it comes in two variants. The promoter of the three segments coding for the polymerase subunits have a C residue at position 4 (C4 promoter), while the promoter of the five remaining segments has a U residue at this position (U4 promoter). Using C4 and U4 promoters, Lee and Seong (1998) showed that the C4 RNA promoter promotes replication and downregulates transcription, and that these two promoters fold differentially (Lee et al., 2003). However, their NMR structural study (Lee et al., 2003) was performed on short RNA oligonucleotides in the absence of proteins and its biological relevance might be limited. Nevertheless, in the crystal structure of the IBV polymerase, the U4 residue in the 3′ strand makes specific contacts with Val133 and Arg135 of the PB1 subunit (Reich et al., 2014), and hence binding to C4 and U4 promoters is likely slightly different, which might have an effect on the transcription/replication switch.

Finally, another mechanism influenced by the vRNA promoter is the recognition of IAV by the host innate immune system. Liu et al. (2015) analyzed the relation between the vRNA promoter of IAV and RIG-I activation. They first demonstrated that transfection of cells with vRNA induced production of IFN in a strain-independent but vRNA dose-dependent manner and that RIG-I is the major actor in the IFN response against IAV. They then mapped the region of the vRNA that is important for RIG-I activation using truncated constructions and showed that the panhandle structure formed by the promoter *in vitro* is sufficient to induce IFN production and more importantly that the degree of complementarity in the proximal step of the promoter is critical for RIG-I activation (Liu et al., 2015). Similarly, Anchisi et al. (2016) observed that the presence of mismatches in the vRNA promoter decreased formation of the vRNA-RIG-I complex and also reduced IFN-B production. Interestingly, the nucleotide composition of the WT promoter region appears to be optimized for a good balance between vRNA and vmRNA synthesis and for limiting RIG-1 activation. Lee et al. (2016) further investigated the role of two distinct features of the promoter, the 5′-PPP moiety proposed to be an agonist of RIG-I (Rehwinkel et al., 2010) and the bending at the 5′-terminal stem, in its recognition by RIG-I. They concluded that the helical bend is more important for the interaction with RIG-I than the 5′-PPP moiety (Lee et al., 2016).

#### The vRNA Internal Region

Until very recently not much was known about the structure of vRNAs, apart from the promoter region. However, several studies addressed this question using *in vitro* or/and bioinformatics approaches or tried to identify regions that are important for the packaging.

Gultyaev et al. (2016) performed a bioinformatics study of the putative conserved secondary structures that might impose evolutionary constraints on the HA vRNA. Covariation analysis indicated that the structured domains in HA vRNA are mostly subtype specific. Several local structured domains that are not essential for viral replication but contribute to viral fitness were identified and were proposed to play a role in virus evolution and re-assortment (Gultyaev et al., 2016). Of note, a similar study on the NP segment identified a functional pseudoknot structure in the packaging signal region of the NP vRNA (Gultyaev et al., 2014).

A bioinformatics study of the RNA structure of segment M in both (+) and (-) strands indicated that the first structure found on both strands is similar, consisting of a stem-loop (nucleotides 219-240), named SL3-10, the difference being that on the (+) strand the loop is 8 nts long, while it is only 4 nts long in (-) strand, but with a longer stem (**Figure 3A**) (Kobayashi et al., 2016). The same type of structures were found around positions 967–994 for both strands, named SL5-3B, with a second stem loop just before in the (+) strand at positions 950–964, named SL5-2 (**Figure 3B**) (Kobayashi et al., 2016). Mutations disrupting SL3-10 and SL5-3B structures impaired viral replication and SL3-10 mutants also produced more defective particles, leading to the hypothesis that these secondary structures are important for packaging (Kobayashi et al., 2016).

Secondary structure models of the NS and M fragments based on RNA chemical mapping experiments and structure predictions were recently published (Lenartowicz et al., 2016; Ruszkowska et al., 2016). They showed that these vRNAs are highly structured with many bulges and internal loops. However, since these structures were obtained *in vitro* in the absence of NP protein and polymerase complex, they most likely do not reflect the actual vRNA structure found within vRNPs in the infected cells or in the viral particles. Nevertheless, the presence of multiple single stranded regions suggests that they could constitute potential interaction sites between the different vRNAs leading to an efficient and specific packaging (Gerber et al., 2014).

Such interactions have been described *in vitro* for a human H3N2 (Fournier et al., 2012a,b) and an H5N2 strain (Gavazzi et al., 2013a). It was observed, using an electrophoretic mobility shift assay, that the 8 vRNAs of each of these strains form a network of interactions, but unexpectedly, these networks are strain specific (Fournier et al., 2012a,b; Gavazzi et al., 2013a). These interactions may be involved in the specific packaging of the influenza virus genome. Indeed, the interaction between segments PB1 and NS of the avian H5N2 virus that was identified *in vitro* has actually been shown to be required for optimal packaging into viral particles (Gavazzi et al., 2013b). The interacting sequences can fold into weakly stable stem-loops, suggesting that the interaction between these two RNAs might initiate via a kissing-loop mechanism (**Figure 4**). To date this remains the only inter-vRNA interaction that has been unambiguously demonstrated to play a role in packaging.

Very recently, additional interactions between the vRNA segments of an H1N1 strain have been proposed using SPLASH (Sequencing of Psoralen crosslinked, Ligated, And Selected Hybrids) (Dadonaite et al., unpublished). Most segments could potentially interact with at least two other vRNAs, and NA and NS are the vRNAs that form the fewest interactions. Mutations aiming at disrupting the proposed intermolecular interactions resulted in packaging defects (Dadonaite et al., unpublished); however, in the absence of *trans*-compensatory mutants the observed defects could also be attributed to alteration of functionally important intramolecular structures. In the same study, SHAPE-MaP experiments (Selective 2′-Hydroxyl Acylation Analyzed by Primer Extension and Mutational Profiling) were performed to compare the flexibility profile of each vRNA nucleotide *in vitro* and *ex vitro*, after removal of the vRNP-associated proteins (Dadonaite et al., unpublished). The results suggest that the structure is more flexible *in vitro* with uneven NP binding along the genome. Interestingly, a recent HITS-CLIP (High Throughput Sequencing of RNA isolated by CrossLinking ImmunoPrecipitation) study showed that NP does not bind evenly along the vRNAs: in each vRNA, there are zones of low NP density (Lee et al., 2017), which might be involved in intermolecular base-pairing. These results were confirmed by a very recent PAR-CLIP study, which also showed that on average a NP monomer binds 12 vRNA nucleotides and that there is a mean distance of 25 nucleotides between adjacent NP binding sites (Williams et al., 2018). Moreover, these authors identified 24 regions with low NP binding, representing about 10% of the genome, with some of them expected to form secondary structures. Mutations designed to disrupt these structures were introduced in segments PB2, PB1, NP, and NS and resulted in packaging defects, whereas mutations preserving the predicted vRNA structure had no effect on vRNA packaging and viral replication (Williams et al., 2018). These results strongly suggest that the predicted vRNA structures within the low NP-binding regions are important for the coordinated packaging of a complete set of eight IAV segments.

Altogether, these recent studies suggest that both intermolecular vRNA-vRNA structures and intramolecular vRNA structures in NP-devoided regions play a major role in the selective packaging process of influenza viruses.

### Positive Polarity RNA

While most of the structural elements that will be described below are specific to cRNA or mRNA, there is a common feature shared by these two molecules. In 2013, a study investigated binding of the NS1 protein to RNA and its specificity using SELEX (Systematic Evolution of Ligands by Exponential enrichment) experiments (Marc et al., 2013). It was shown that both the sequence and the structure were important. The protein recognizes the AGCAAAAG motif that is present in both mRNA and cRNA in all IAV segments (Marc et al., 2013). Moreover, this binding depends on the structure of this motif, since minor changes such as deletion of a 1-nt bulge is sufficient to highly reduce binding (Marc et al., 2013). This structural feature might be important in the viral life cycle for allowing NS1 to play its roles in translation and nucleocytoplasmic transport.

#### Complementary RNA (cRNA)

Unlike vmRNAs, cRNAs have a terminal promoter that is required for vRNA synthesis and is assumed to be organized in the same way as the vRNA promoter. However, due to the imperfect complementarity between the 5′ and 3′ RNA termini, the vRNA and cRNA promoters must be slightly different. Comparing the NMR structure of short vRNA and cRNA promoters in the absence of polymerase, Park et al. (2003) observed that the terminal region of the cRNA panhandle is extremely unstable. However, by analogy with the vRNA promoter, it is likely that the cRNA promoter does not adapt a panhandle structure when bound to the polymerase complex (see section “The Terminal Promoter”).

**FIGURE 5:**
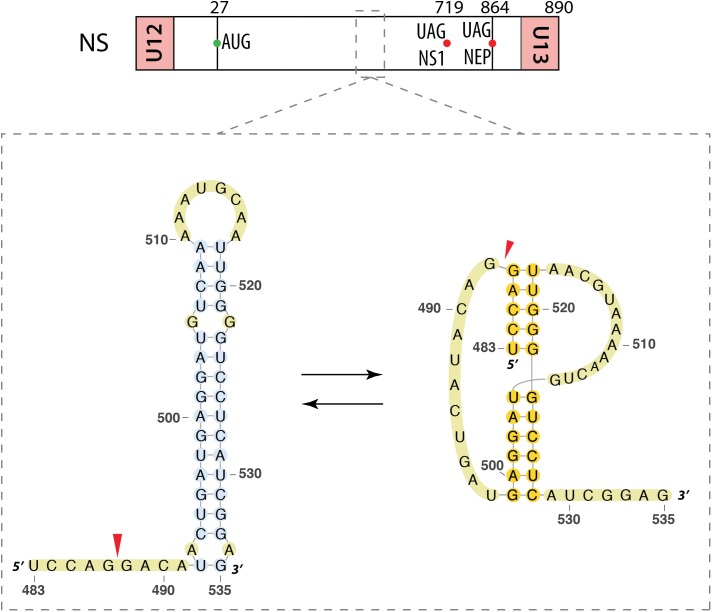
Alternative secondary structures at the 3′ splice site of the segment NS mRNA. Their locations are indicated by a dashed square on the segment scheme at the top of the figure. The red arrow indicates the 3′ splice site.

In the historical corkscrew model of the vRNA promoter, nucleotide A10′ is single stranded and forms a flexible hinge between the short 5′ stem-loop and the distal stem (**Figure 1B-3**), whereas in the cRNA promoter A10′ can form a base pair with U11, while U10 is unpaired (**Figure 1B-5**). It was proposed that these differences between the promoters induce a different recognition by the polymerase complex (Flick et al., 1996) and allow discrimination between vRNA and the cRNA (Tchatalbachev et al., 2001). Indeed, while both cRNA and vRNA form RNPs, only the latter is found within virions. Based on the biological activity of promoter mutants, Tchatalbachev et al. (2001) suggested that an unpaired nucleotide at position 10′ is required for RNP export and hence packaging; they therefore proposed an interaction between M1 and the polymerase complex rather than NP to explain their results (Tchatalbachev et al., 2001). However, in contrast with these results, a recent study reported that vRNPs and cRNPs are both efficiently exported from the nucleus, but only vRNPs traffic to the plasma membrane, indicating that discrimination occurs during cytoplasmic transport (Chaimayo et al., 2017).

**FIGURE 6:**
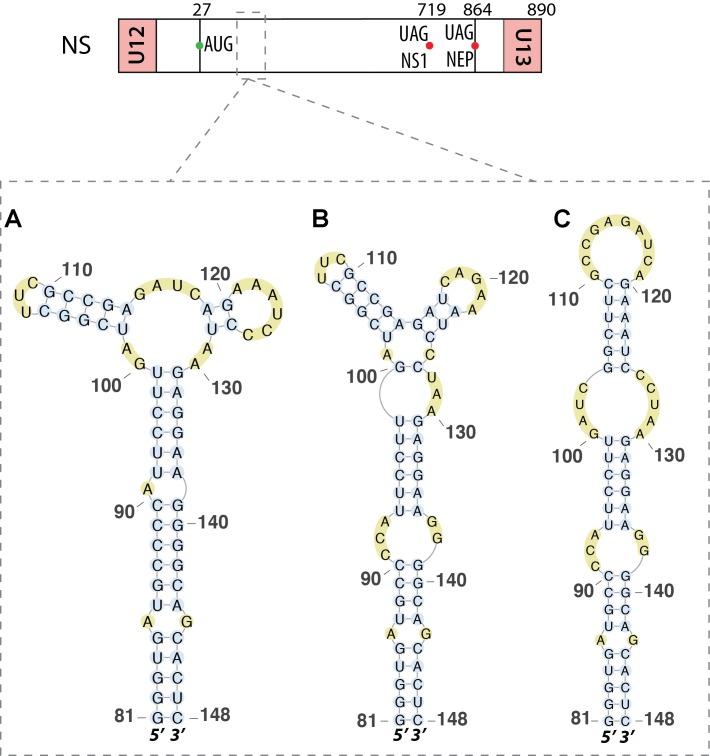
Different secondary structures proposed for the 5′ splice site of segment NS mRNA. Their locations are indicated by dashed squares on the segment scheme at the top of the figure. **(A)** The multi-branch structure proposed by Ilyinskii et al. (2009). **(B)** The tetraloop hairpin model presented by Moss et al. (2011). **(C)** The hairpin structure proposed and validated by chemical probing experiments by Priore et al. (2013a).

If we consider the X-ray crystallographic structure of the vRNA promoter in complex with the viral polymerase (Pflug et al., 2014; Reich et al., 2014), the cRNA promoter can form the same 5′ hook structure, including the two non-canonical A-A base pairs (**Figure 1B-6**) that plays a key role in the recognition of the polymerase complex. However, the distal stem of the cRNA promoter is one base pair shorter than its vRNA counterpart, its 3′ single-stranded region is one nucleotide longer (due to the unpaired U11), and the single-stranded nucleotides 3, 5, and 8 are different. Interestingly, nucleotides at positions 3 and 8 interact in a base-specific manner with the viral polymerase (Reich et al., 2014) and one can therefore expect than the vRNA and cRNA promoters interact with the polymerase complex in slightly different ways. This is corroborated by the fact that nucleotides 2–5 and 7–9 of the cRNA promoter are essential for activity (Crow et al., 2004). These authors also found that the 5′ hairpin, but not the 3′ hairpin, of the corkscrew model is required for the activity of the cRNA promoter, and overall their results are in good agreement with the recently visualized hook model (Crow et al., 2004).

In 2012, a study looked at Global Ordered RNA Structure (GORS) in the whole Influenza genome (both strand orientations). GORS revealed ‘excess’ thermodynamic stability of wild-type RNA sequences versus random RNA of the same composition. This study demonstrated that GORS only exist in the (+) strand of segments PB2, NP, and NS (Priore et al., 2012) and that these GORS evolved in a host specific way. At the moment, the biological significance of these GORS remains to be demonstrated.

#### Viral Messenger RNA (vmRNA)

The vRNA and cRNA promoters are conserved in all segments of all influenza strains and thus their structure attracted a lot of attention. However, influenza A vmRNAs might also contain segment-specific structural motifs that regulate processes such as translation, export, or splicing. The later process, which is required for production of the NS2 and M2 proteins, has been the subject of several studies.

##### Segment NS

Gultyaev et al. (2007) focused on the segment NS vmRNA. Using a bioinformatics approach, they identified a region conserved between different strains of IAV and also in an IBV strain (nucleotides 524–574 for IAV and 716–767 for IBV). This region is predicted to form a hairpin structure, which is more stable in the IAV strains that emerged recently (Gultyaev et al., 2007). They also found that this region can adopt an alternative pseudoknot structure (**Figure 5**). These results were supported by an independent bioinformatics analysis (Moss et al., 2011). Interestingly, native gel electrophoresis revealed no evidence of the pseudoknot conformation in a strain that presents the stabilized hairpin structure. Considering the location of this structure in the segment NS pre-mRNA (**Figure 5**), it was suggested that the equilibrium between the two structures could regulate splicing. Indeed, a single mutation predicted to induce misfolding of this RNA region strongly reduced production of the NS2/NEP mRNA and attenuated viral replication, whereas introduction of a second site mutation predicted to restore RNA folding restored splicing and viral replication, demonstrating the importance of the folding of this RNA region for splicing regulation (Jiang et al., 2016).

Ilyinskii et al. (2009) analyzed a region located near the 5′ end (nucleotides 81–148) of the same vmRNA. They found that this region folds into a multi-branch structure (**Figure 6A**). Disruption of this secondary structure reduced transcription of the NS1 vmRNA. However, this structure was questioned by Moss et al. (2011) who proposed an alternative four loop hairpin model for this region (**Figure 6B**), again using a bioinformatics approach. Priore et al. (2013a) used structure prediction software to discover a third structure that was predicted to fold as a single extended hairpin (**Figure 6C**). They used RNA chemical probing and isoenergetic microarray techniques to confirm that this hairpin structure exists in the mRNA *in vitro* (Priore et al., 2013a). A recent bioinformatics prediction confirmed earlier findings in this region, with an extended hairpin structure conserved among all human and non-human IAVs and derived from the A/Brevig_Mission/1/1918 (H1N1) strain (Vasin et al., 2016). This study also predicted a hairpin structure derived from the same pandemic H1N1_1918_ strain between nucleotides 497 and 564 of this RNA (Vasin et al., 2016).

##### Segment M

**FIGURE 7:**
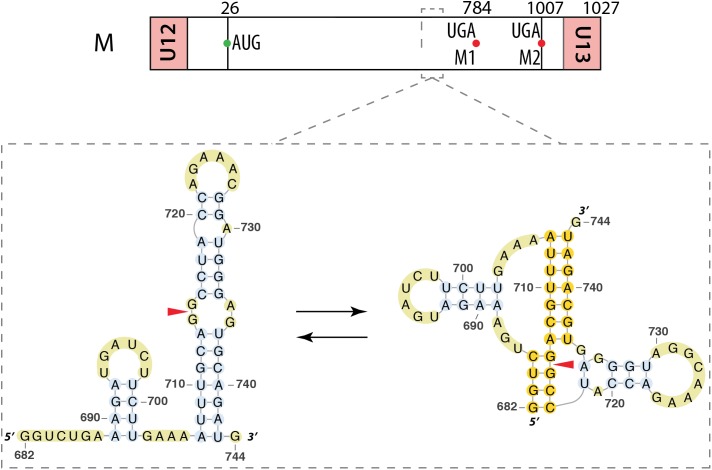
Alternative secondary structures at the 3′ splice site of the segment M mRNA. Their locations are indicated by a dashed square on the segment scheme at the top of the figure. The red arrowhead indicates the splice site.

**FIGURE 8:**
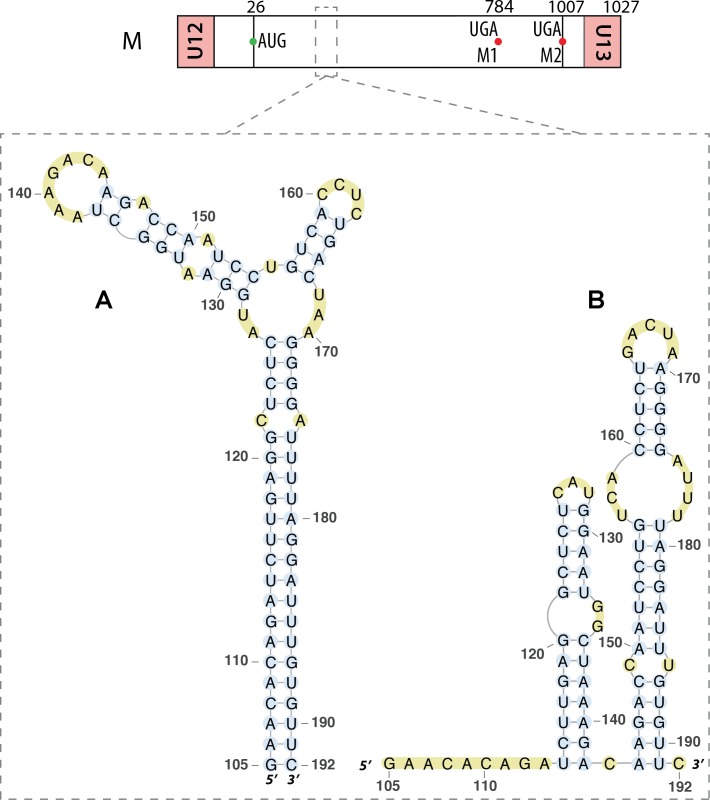
Different secondary structures proposed for the 5′ splice site of segment 7 mRNA. Their locations are indicated by a dashed square on the segment scheme at the top of the figure. **(A)** Multi-branch structure proposed by Moss et al. (2011). **(B)** Double hairpin structure proposed by Jiang et al. (2014).

Segment M also requires a splicing event to allow expression the M2 ion channel protein. Interestingly, the region surrounding the 3′ splice site of this segment (nucleotides 714–715) displays the same structural feature as the 3′ splice site of segment NS, namely an equilibrium between a pseudoknot and a double hairpin structure that was first proposed by bioinformatics analysis (**Figure 7**) (Moss et al., 2011) and supported by native gel electrophoresis, chemical and enzymatic RNA probing, and oligonucleotide binding (Moss et al., 2012a), as well as further sequence analysis (Moss et al., 2012b). As for segment NS, the equilibrium between these structures is thought to play a role in the splicing regulation. The structure of the larger hairpin was determined by NMR and, due to its unique structural features, including a base-triple, it was suggested to constitute a potential therapeutic target (Chen et al., 2015). A region downstream of the 5′ splice site of the segment M pre-mRNA (nucleotides 105–192) was also investigated. A multi-branch structure model was predicted by bioinformatics (**Figure 8A**) (Moss et al., 2011). This region can also adopt an extended hairpin conformation (**Figure 8B**) but chemical and enzymatic RNA mapping, mutagenesis and NMR all support the multi-branch structure (Jiang et al., 2014). However, mutants designed to induce misfolding of the RNA structure of the regions close to the 3′ splice site and downstream of the 5′ splice site have little effect on the production of the M1 and M2 proteins, even though they have a small effect on viral replication (Jiang et al., 2016).

##### Segment NP

**FIGURE 9:**
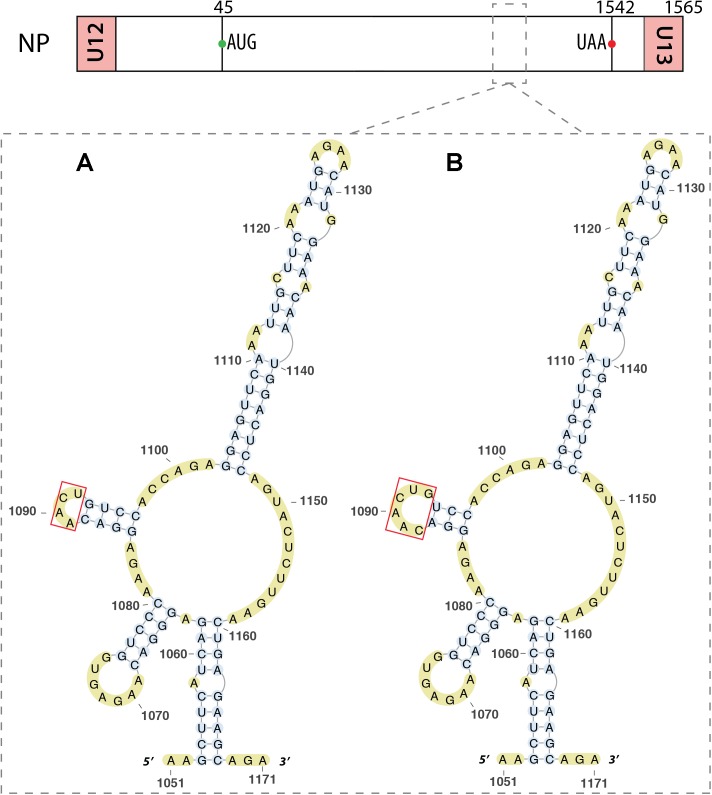
RNA secondary structure in the mRNA of segment 5. Predicted **(A)** and experimental **(B)** structures are almost identical. The red box shows the only difference, located in a loop, between these two structures. The region of interest is indicated by dashed square on the segment scheme at the top of the figure.

Within the NP vmRNA, nucleotides 1051–1171 were predicted to be structured (Moss et al., 2011; Soszynska-Jozwiak et al., 2015). Indeed, Soszynska-Jozwiak et al. (2015) proposed that this region folds into a multi-branch structure with three hairpins (**Figure 9A**), that they slightly revised based on chemical and enzymatic probing experiments (**Figure 9B**).

##### Segment PB1

Another pseudoknot was predicted on the PB1 vmRNA (nucleotides 65–126); it contains the start codon of two alternative polypeptides encoded by this segment, PB1-F2 and N40. This structure was predicted using bioinformatics (Moss et al., 2011) and confirmed using chemical probing experiments, although another structure was also proposed in this region consisting of two hairpins separated by a single stranded region (Priore et al., 2015). The pseudoknot structure is observed in presence of Mg^2+^ and has been proposed to regulate translation of PB1-F2 and N40.

## Influenza B and C Viruses

The polymerase complex of Influenza B virus bound to the vRNA promoter was crystalized and its X-ray structure was determined. Indeed, the IBV polymerase binds the vRNA promoter in the same way as the IAV polymerase and the IBV vRNA promoter also adopts the hook conformation when bound to the viral polymerase (Reich et al., 2014). Regarding ICV, only the apo polymerase complex was crystalized, and a comparison of the structures of the influenza polymerases with and without a bound vRNA promoter suggests that the apo polymerase is in a “closed-state” that is activated upon binding of the promoter (Hengrung et al., 2015).

To determine possible structural constrains acting on Influenza viruses, protein-coding sequences of IBV were used to perform a survey for conserved secondary structure in the (+) and the (-) RNAs. The GORS analysis revealed similarities between IAV and IBV and GORS have been found in both RNA orientations in NP, NS, and PB2 segments (Priore et al., 2013b). In general, influenza B RNA motifs are more stable in the (+) RNA than in the (-) RNA, except for the HA fragment. Some differences also exist between IAV and IAB as segments M and PB1 display GORS only in IAV and IBV, respectively.

Previous studies on IAV described a role for RNA structural motifs in the splicing of M and NS vmRNAs. The corresponding regions of IBV and ICV genomes have been scanned with the RNAz program and regions of high thermodynamic stability and conserved RNA secondary structure overlapping the 5′ splice sites of IBV segment NS and ICV segments M and NS were detected. In contrast, the structural motifs detected close to the IAV 5′ splice sites of segments M and NS do not overlap with these sites (Dela-Moss et al., 2014).

Segment 8 (NS) of IAV is homologous to segments 8 and 7 of IBV and ICV, respectively, and in all cases NS2/NEP mRNA is produced by splicing. In both IBV and ICV, the 5′ splice site is located into the stem of a conserved hairpin, possibly sequestering the splice site. Additionally, a conserved hairpin is also predicted specifically in segment 6 (M) of ICV, in which the M1 protein is produced by splicing, in contrast to IAV and IBV, in which M1 is translated from the unspliced vmRNA (Dela-Moss et al., 2014). Moreover, in both IBV and ICV, the 3′ splice sites of the two shortest segments can fold either as a hairpin or as a pseudoknot, changing the accessibility of the splice site, as also described for IAV (Gultyaev et al., 2007; Moss et al., 2011, 2012a). Interestingly, naturally occurring mutations in regions bordering the splice sites preserve base pairing (Dela-Moss et al., 2014).

## Conclusion

In this review, we focused on the relation between the RNA structure of influenza viruses and its function in the replication cycle. Most of our knowledge about influenza RNA structure and function deals with the vRNA/cRNA promoters and the splice sites of vmRNAs. Concerning the promoter, the recent X-ray structures of the viral heterotrimeric polymerase alone and bound to the vRNA promoter (Pflug et al., 2014; Reich et al., 2014; Hengrung et al., 2015) shed a new light on previous functional studies, but further studies will be required to fully understand the viral replication and transcription mechanism. Given their key functional roles and their conservation among all segments of all IAV strains, vRNA and cRNA promoters might be good targets for the development of anti-IAV drugs. Several studies already investigated this possibility. A compound named DPQ [6,7-dimethoxy-2-(1-piperazinyl)-4-quinazolinamine] was found to bind the vRNA promoter and induces important changes in its 1D NMR imino resonance spectrum. In cell-based assays, DPQ reduces replication of IAV and IBV but it is not as potent as FDA-approved drugs (Lee et al., 2014). However, more potent derivatives of DPQ have been identified that pave the way for the design new drugs (Bottini et al., 2015).

Concerning the splice sites, several different structures have been proposed based on bioinformatics predictions. In several cases, chemical and enzymatic mapping experiments helped identify the actual structure. Interestingly, the IAV 3′ splice site of both segment M and NS mRNAs share the same structural feature, which has been shown to regulate splicing and viral replication. By contrast, the M and NS 5′ splice sites adopt different structures.

Until recently, our knowledge of the structure of the internal part of vRNAs within vRNPs was almost inexistent. However, rapid progress in this area has been made within the last months thanks to the coupling of several classical RNA biochemistry tools with next generation sequencing (Lee et al., 2017; Williams et al., 2018; Dadonaite et al., unpublished). These studies identified NP-rich and NP-poor regions in each vRNAs (Lee et al., 2017; Williams et al., 2018) and obtained genome wide structural information (Dadonaite et al., unpublished). Several intramolecular structures and intermolecular RNA–RNA interactions have been proposed in these studies (Williams et al., 2018; Dadonaite et al., unpublished), which open the way for new functional and structural studies.

## Author Contributions

DF, MC, AP-S, RPS, J-CP, and RM conceived the review topic. DF, MC, and RM drafted the manuscript and DF generated the figures. AP-S, RPS, and J-CP corrected and edited the manuscript. All authors read and approved the final version of the manuscript.

## Conflict of Interest Statement

The authors declare that the research was conducted in the absence of any commercial or financial relationships that could be construed as a potential conflict of interest. The handling Editor declared a shared affiliation, though no other collaboration, with the authors DF, MC, AP-S, RS, J-CP, RM.
